# Prevalence of multiple micronutrient powders consumption and its determinants among 6- to 23-month-old children in East Africa: a mixed effect analysis using the recent population based cross sectional national health survey

**DOI:** 10.1186/s40795-024-00888-0

**Published:** 2024-05-31

**Authors:** Bewuketu Terefe, Bogale Chekole

**Affiliations:** 1 Department of Community Health Nursing, School of Nursing, College of Medicine and Health Sciences, University of Gondar, Post Office Box: 196, Gondar, Ethiopia; 2 Department of Comprehensive Nursing, College of Medicine and Health Sciences, Wolkite University, Southern, Ethiopia

**Keywords:** Prevalence, Multiple micronutrient powders, Determinants, 6–23 months old children, East Africa

## Abstract

**Background:**

To address iron deficiency anemia, Multiple Micronutrient Powders (MMNPs) can be sprinkled onto any semisolid diet and given to young children. There is currently no data on actual MMNPs uptake by children; hence, the study’s goal was to investigate MMNPs and determinants among children aged 6–23 months in East Africa.

**Methods:**

Data from the 2016–2022 East Africa demographic and health survey extracted from Kids Records (KR) files were used in this study. A total of 33,324 weighted 6- to 23-month-old child samples were included. For assessing model fitness and contrast, the intra-class correlation coefficient, median odds ratio, proportional change in variance, and deviance were used. A multilevel logistic regression model was applied to identify variables that may influence MMNPs intake. In the multivariable multilevel logistic regression analyses, variables were judged to be significantly linked with MMNPs intake if their *p*-values were < 0.05.

**Results:**

In East Africa, the prevalence of MMNPs intake among infants aged 6–23 months was 6.45% (95% CI, 6.19%, 6.22%). Several factors were found to be significantly associated with MMNPs consumption. These factors include older maternal age (AOR = 1.23, 95% CI, 1.09, 1.39) and (AOR = 1.46, 95% CI, 1.23, 1.73), poorer (AOR = 0.73, 95% CI, 0.64, 0.84), middle (AOR = 0.75, 95% CI, 0.66, 0.86), richer (AOR = 0.61, 95% CI, 0.52, 0.71), and richest (AOR = 0.49, 95% CI, 0.41, 0.59) as compared to poorest, having employment status (AOR = 0.65, 95% CI, 0.59, 0.71), mass media exposure (AOR = 1.61, 95% CI, 1.35, 1.78), longer birth interval (AOR = 1.19, 95% CI, 1.28, 1.36), place of delivery (AOR = 1.46, 95% CI, 1.28,1.66), and mothers from rural areas (AOR = 0.71, 95% CI, 0.62,0.80).

**Conclusions:**

Overall, MMNPs intake was lower than the national and international recommendations. Only seven out of every hundred children received MMNPs. Improving maternal preventive health care and supporting marginalized women will have a positive impact.

## Introduction

The use of several micronutrient powders for point-of-use fortification of foods has been proposed by the World Health Organization (WHO) as an option for mitigating or eliminating the limits associated with supplementation and mass fortification. They are designed to boost the vitamin and mineral intake of infants and toddlers aged 6 to 23 months [1]. The use of various micronutrient powders is a population-level preventive technique that does not require screening for any ailment or disease. While micronutrient powders have been found to be effective in reducing anemia and iron deficiency among children and increasing their intake of vitamins and minerals, children with anemia should still be treated according to the guidelines provided by the WHO, and national guidelines [1, 2].

However, research suggests that multiple micronutrient (MN) deficiency in children is recognized as a global public health problem, with low- and middle-income countries faring the worst [3, 4]. It is difficult to estimate global micronutrient deficiencies in children under the age of two, although it has been estimated that 190 and 293 million preschool children, respectively, had vitamin A insufficiency and anemia [5, 6]. According to the 2019 report of the United Nations Children’s Fund, over 340 million children globally suffered from hidden hunger caused by MN deficiencies [7]. The problem is significantly worse in low-and middle- income countries, with limited empirical research showing that just 29% of toddlers aged 6–23 months in Ethiopia were provided the minimum diversified diet (MDD) in 2018 [8]. Iron, zinc, calcium, iodine, manganese, chromium, copper, fluoride, and vitamins are among the important MNs required for life [9, 10]. Although MNs are only required in trace amounts, their removal from the diet has a deleterious impact on children’s survival and development. Furthermore, the MN deficiency has devastating effects such as stunting, wasting, weakened immunity, and delayed cognitive development [11–13].

Individual and community-level factors linked with MN intake include mothers’ sociodemographic and child features, dietary habits, community-level lifestyle, and site of residence [14–16]. In addition to the aforementioned characteristics, the usage of maternal healthcare services such as antenatal care (ANC), institutional birth, and postnatal care (PNC) is linked to children’s MN intake status [17, 18]. Most East African countries have poor infrastructure and inaccessible health services, as well as limited access to health facilities [19, 20]. Individual and community-level factors linked with MN intake in children, on the other hand, are rarely studied. Using the most recent DHS data, this study aims to examine MN consumption status and related variables among children aged 6–23 months in East Africa. In Eastern African locations, there is a scarcity of published evidence regarding MN intake among children aged 6–23 months. The findings could provide valuable insights for developing contextual methods for problem mitigation and serve as baseline data for present practice.

## Methods

### Data sources and sampling procedures

We analyzed the most recent Demographic and Health Survey (DHS) data from nine East African nations (Burundi, Ethiopia, Kenya, Madagascar, Malawi, Rwanda, Tanzania, Uganda, and Zambia) collected between 2016 and 2022 to determine the practice of receiving MMNPS and the factors that influence it in East Africa. These current datasets were combined in order to investigate the prevalence and associated determinants of various micronutrient powders use among children aged 6–23 months in Eastern Africa.

The DHS surveys are collected on a five-year cycle in low- and middle-income countries using structured, pretested, and validated questionnaires. The DHS surveys use the same standard sampling, questionnaires, data collection, and coding procedures, allowing for multi-country analysis. A stratified, two-stage cluster sampling technique is used in the DHS survey. Clusters and enumeration areas (EAs) were randomly picked from the sample frame in the first stage (they are typically created from the most recent national census). The second stage involved systematic sampling of households in each cluster or EA. Interviews were done with target demographics (women aged 15–49 and males aged 15–64) in selected houses. This study included all children aged 6–23 months who received MMNPs in the six months preceding the most recent DHS of nine East African nations. According to the DHS Statistics Guide, missing values and “don’t know” are omitted from the numerator (assuming they did not consume or receive). Any missing data at any outcome variable was managed using various missing data management procedures in accordance with the DHS statistics guide’s instructions [21]. A total of 33,324 weighted young infants aged 6 to 23 months were involved in the study (Table 1).

**Table 1:** Countries, sample size, and survey year of demographic and health surveys included in the analysis for 12 East African countries

Country	Survey year	Sample size(weighted)	Frequency(weighted)
Burundi	2016/17	4,086	12.26
Ethiopia	2016	3,048	9.15
Kenya	2022	5,100	15.30
Madagascar	2021	3,527.5108	10.59
Malawi	2016/17	4,878	14.64
Rwanda	2019/20	2,456	7.37
Tanzania	2015/16	3,101	9.31
Uganda	2016	4,369	13.11
Zambia	2018	2,760	8.28

### Data management and statistical analysis

STATA version 17 was used to extract, clean, and recode the study’s variables. During any statistical analysis, the data was weighted using sample weight to account for the differential probability of selection due to the sampling strategy used in DHS data. As a result, the survey findings were guaranteed to be representative. To account for the hierarchical nature of the data, a two-level mixed effect univariate and multivariable logistic regression analysis were employed to evaluate the effect of explanatory variables on the consumption of various micronutrient powders. The data is divided into two levels, with a group of J EAs and within-group j (j = 1, 2…J), and a random sample nj of level-one units (6–23 months old children). The response variable is represented as; Y_ij_ = 0 if the i^th^ children was in the j^th^ EAs had not a history of MMNPs consumption 1 if i^th^ children was in the j^th^ EAs had history MMNPs consumption.

To account for the nested effect, acceptable deductions and conclusions from this data require adequate modeling techniques, such as multilevel modeling, which includes variables assessed at multiple levels of the hierarchy [22]. For the data, four models were fitted. To calculate the extent of cluster variation in MMNPs, the first model was an empty one with no explanatory factors. Intra-class correlation coefficients (ICC), proportional change in variance (PCV), and median odds ratio (MOR) were used to calculate differences between clusters (EAs). The ICC is the proportion of variance explained by the population grouping structure. In contrast to the null model, PCV evaluates the overall variation ascribed to individual and community-level components in the multilevel model [23].

The MOR is also defined as the median value of the odds ratio between the clusters at high and low risk of multiple micronutrient consumption when two clusters (EAs) are chosen at random. The second model was fitted with only community-level variables, the third with only individual-level variables, and the fourth with both individual and community-level variables. The deviation (-2LLR) of these four models was evaluated, and the model with the lowest deviance was chosen as the best-fitted model for the data.

In the bivariable analysis, variables with a *p*-value of ≤ 0.2 were considered for the multivariable analysis. The best-fitted model’s adjusted odds ratio (AOR) with 95% CI was provided in the multivariable multilevel binary logistic model to find the associated factors of MMNPs uses. The final model’s statistical significance was set at *p* < 0.05. In the bivariable analysis, variables with a *p*-value of ≤ 0.2 were considered for the multivariable analysis. The best-fitted model’s adjusted odds ratio (AOR) with 95% CI was provided in the multivariable multilevel binary logistic model to find the associated factors of MMNPs uses. The final model’s statistical significance was set at *p* < 0.05.

### Variance inflation factor analysis

Before going to the analysis section, each dependent variable was assessed regarding its variance, inflation factors, and tolerances. The overall mean VIF of this study was 1.61.

### Variables of the study

#### The outcome variable

The outcome variable of this study was the number of living children aged 6–23 months who received MMNPs in the seven days preceding the interview. Then the outcome variable was recategorized as Yes = “1” if the child received MMNPs and No = “0” if the child did not receive them. This classification and the analysis was made according to the guide to the DHS statistics book [21]. .

#### The independent variables

Based the previous literature various maternal and child-related factors were included [8, 10, 16, 24, 25]. This included maternal age, educational status, types of place of residence, marital status, household wealth index, current employment status, mass media exposure, Antenatal Care (ANC) follow-up, place of delivery, Postnatal Care (PNC) checkup, number of health visits, total children born, under-five children, age of the child, sex of the child, size at birth, twin status, birth order, preceding and succeeding birth interval in months, sex of the household head, community level literacy, ANC, and community level place of delivery.

### Consideration of ethics

This study is a secondary data analysis of DHS data from nine East African nations (Burundi, Ethiopia, Kenya, Madagascar, Malawi, Rwanda, Tanzania, Uganda, and Zambia); hence, no ethical approval is required. This study’s online registration and request for measures of DHS were carried out. After receiving permission to access the data, the dataset was obtained from the DHS online archive (http://www.dhsprogram.com).

## Results

### Sociodemographic characteristics of the study participant

In this study, a total of 33,324 children whose ages ranged from 6 to 23 months were enrolled in East African countries. Nearly half (44.69%) of the study women were between 25 and 34 years of reproductive age. Regarding marital status, the majority of mothers (22,346 or 67.06%) were married. In terms of place of residence types, 26,270 (78.83%) of mothers resided in rural areas. With regards to educational status, 16,932 (50.81%) had a primary level of education. Regarding wealth index, 7,837 (23.52%) of mothers belonged to the poorest households. Additionally, 25,716 (77.17%) of mothers had institutional deliveries, and 31,593 (94.81%) had at least one ANC follow-up during their pregnancies. Similarly, about 17,958 (53.89%) and 21,749 (65.27%) of women had at least one mass media exposure (either listening to radio, watching television, or reading magazines or newspapers), respectively. Furthermore, about 16,756 (50.28%) and 23,007 (69.04%) participants had preceding birth intervals of 24–59 months and succeeding birth intervals of more than 60 months, respectively (Table 2).

**Table 2:** Sociodemographic, maternal and child related characteristics on multiple micronutrient powder consumption among 6–23 months old in east African countries recent DHS (*n* = 33,324, and unweighted *n* = 33,647)

Multiple micro nutrient powder	Frequency(weighted)	Percentage
**Variables**		
**Maternal age**		
15–24	12,049	36.16
25–34	14,894	44.69
35–49	6,381	19.15
**Maternal education**		
Not educated	6,887	20.67
Primary	16,932	50.81
Secondary & higher	9,505	28.52
**Maternal employment**		
No	11,575	34.73
Yes	21,749	65.27
Wealth index		
Poorest	7,837	23.52
Poorer	7,022	21.07
Middle	6,466	19.40
Richer	6,125	18.38
Richest	5,875	17.63
**Mass media exposure**		
No	17,958	53.89
Yes	15,366	46.11
**Preceding birth interval in months**		
7–24	5,783	17.35
24–59	16,756	50.28
> 60	10,785	32.36
**Succeeding birth interval in months**		
7–24	3,653	10.96
24–59	6,664	20.00
> 60	23,007	69.04
**Total children born**		
< 3	14,904	44.72
3–5	12,479	37.42
> 5	5,941	17.83
**Marital status**		
Unmarried	10,978	32.94
Married	22,346	67.06
**Place of delivery**		
Home	7,609	22.83
Health facility	25,716	77.17
**ANC follow-ups**		
No	1,731	5.19
Yes	31,593	94.81
**Number health visits**		
Once	25,526	76.60
More than one	7,798	23.40
**Twin status**		
Single	32,460	97.41
Multiple	864	2.59
**Birth order**		
1st	8,364	25.10
2nd or 3rd	12,163	36.50
4th or 5th	6,955	20.87
> 5th	5,842	17.53
**Child size (** ***n*** ** = 30,805)**		
Small	5,500	17.85
Average	16,509	53.59
Large	8,796	28.55
**Number of under five children**		
No	629	1.89
1–2	27,764	83.31
> 2	4,932	14.80
**Place of residence**		
Urban	7,054	21.17
Rural	26,270	78.83
**Community ANC coverage**		
Low	18,112	54.35
High	15,212	45.65
**Community literacy**		
Low	9,739	29.23
High	23,585	70.77
**Community facility delivery**		
Low	25,687	77.08
High	7,637	22.92

In addition, concerning child-related characteristics, the majority of mothers (27,764 or 83.31%) had 1–2 under-five children. Furthermore, almost all children were born as single births (97.41%). Regarding birth order, about 12,163 (36.50%) were 2nd or 3rd birth order, and average birth weight was 16,509 (53.59%). About 12,479 (37.42%) of mothers had approximately 3–5 children in their house (Table 2).

### Prevalence of MMNPs consumption among children

**Fig. 1:**
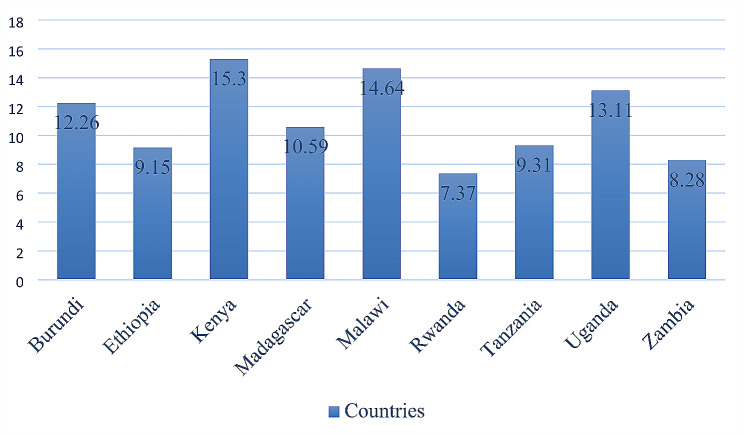
Prevalence of MMNPs consumption across countries among 6–23 months old children in East Africa from 2016–2022

The study revealed that the overall weighted prevalence of MMNPs consumption among children aged 6–23 in East Africa was 6.45% (95% CI, 6.19, 6.72). Among the countries examined, Kenya had the highest prevalence at 15.30% (95% CI, 14.92, 15.69), while Rwanda had the lowest prevalence at 7.37% (95% CI, 7.09, 7.65) (Fig. 1).

### Random effect and factors associated with MMNPs consumption among children aged 6–23 in East Africa

In the null model, there was a significant variance in the probability of being exposed to MMNPs consumption among children in East Africa (community level variance = 0.48, p 0.001). As implied by the intra-cluster correlation coefficient (ICC) in the empty or null model, region differences would account for 12.76% of the variation in children’s MMNPs consumption. Furthermore, the median odds ratio (MOR) was 1.93 (1.80, 2.02). This can be interpreted as meaning that when children go from a low to a high MMNPs consumption or intake prevalence area, the likelihood of being exposed to MMNPs consumption is 1.93 times higher. The PCV of this study was 48.12%, which indicates both community/country-level and individual-level variables explained 48.12% of the national variation observed in an empty model. Determinants such as maternal age, wealth index, marital status, preceding birth interval, employment status, mass media exposure, place of delivery, number of health visits, status of twins, and type of place of residence were statistically significant in the multilevel multivariable logistic regression model among children aged 6–23 months in East Africa.

The odds of being exposed to MMNPs among children increased by 23% and 46%, respectively (AOR = 1.23, 95% CI, 1.09,1.39), and (AOR = 1.46, 95% CI, 1.23,1.73), among women whose age is from 24 to 34 years and from 35 to 49 years old, as compared to women whose age is from 15 to 24 years old. Regarding household wealth index mothers who came from poorer (AOR = 0.73, 95% CI, 0.64, 0.84), middle (AOR = 0.75, 95% CI, 0.66, 0.86), richer (AOR = 0.61, 95% CI, 0.52, 0.71), and richest households (AOR = 0.49, 95% CI, 0.41, 0.49) showed a lower odd of providing MMNPs to their children as compared to poorest household wealth index mothers respectively. Those mothers who are employed have a 45% lower likelihood of providing MMNPs to their children (AOR = 0.65; 95% CI: 0.59–0.71) as compared to unemployed mothers. Women who had mass media exposure showed a higher likelihood (AOR = 1.61; 95% CI = 1.45–1.78) of providing MMNPs to their children as compared to their counterparts. Similarly, mothers who have a longer 60-month and longer preceding month interval revealed a higher probability of providing MMNPs (AOR = 1.19, 95% CI, 1.05–1.36) times to their children as compared to mothers who have a shorter two-year birth interval. Women who gave birth to their children at health facilities showed an odds ratio of 1.46 (95% CI, 1.28–1.66) to expose their children to MMNPs as compared to mothers who gave birth at home. Women who had more than one-time health facility visits in the year had an AOR of 1.12 (95% CI: 1.12–1.24) as compared to women who had only one-time health facility visits. Multiple children have been received 53% more times as compared to single children, by the odds of (AOR = 1.47, 95% CI, 1.15–1.87). Mothers who have come from rural areas showed a lower likelihood of exposing their children to MMNPs (AOR = 0.71, 95% CI 0.62–0.80) as compared to their counterparts. (Table 3).

**Table 3:** Individual and community-level factors associated with multiple micronutrient powder consumption among 6–23 months old in east Africa (weighted *n* = 33,324, and unweighted *n* = 33,647)

Multiple micro nutrient powder	Null model	Model I	Model II	Model III
**Variables**	**AOR (95%CI)**	**AOR (95%CI)**	**AOR (95%CI)**	**AOR (95%CI)**
**Maternal age**				
15–24		1		1
25–34		1.24(1.10,1.41)		1.23(1.09,1.39) *
35–49		1.47(1.24,1.75)		1.46(1.23,1.73) *
**Maternal education**				
Not educated		1		1
Primary		1.03(0.91,1.17)		1.03(0.91,1.17)
Secondary & higher		1.20(1.02,1.40)		1.17(0.99,1.37)
Wealth index				
Poorest		1		1
Poorer		0.73(0.64,0.84)		0.73(0.64,0.84) *
Middle		0.77(0.67,0.88)		0.75(0.66,0.86) *
Richer		0.67(0.56,0.77)		0.61(0.52,0.71) *
Richest		0.61(0.52,0.71)		0.49(0.41,0.59) *
**Maternal employment**				
No		1		1
Yes		0.63(0.58,0.69)		0.65(0.59,0.71) *
**Mass media exposure**				
No		1		1
Yes		1.63(1.47,1.80)		1.61(1.45,1.78) *
**Preceding birth interval in months**				
< 24		1		1
24–59				1.06(0.94,1.21)
> 60				1.19(1.05,1.36) *
**Total children born**				
< 3		1		1
3–5		0.92(0.82,1.04)		0.93(0.82,1.04)
> 5		0.84(0.71,1.01)		0.86(0.72,1.02)
**Marital status**				
Unmarried		1		1
Married		0.90(0.82,0.99)		0.91(0.82,1.00)
**Place of delivery**				
Home		1		**1**
Health facility		1.50(1.32,1.70)		1.46(1.28,1.66) *
**ANC follow-ups**				
No		1		1
Yes		1.22(0.95,1.57)		1.19(0.93,1.54)
**Number health visits**				
Once		1		1
More than one		1.13(1.02,1.25)		1.12(1.02,1.24) *
**Twin status**				
Single		1		1
Multiple		1.48(1.16,1.88)		1.47(1.15,1.87) *
**Place of residence**				
Urban			1	1
Rural			0.71(0.64,0.79)	0.71(0.62,0.80) *
**Community ANC coverage**				
Low			1	1
High			1.18(1.03,1.35)	1.09(0.95,1.24)
**Community illiteracy**				
Low			1	1
High			0.99(0.86,1.13)	1.04(0.91,1.19)
**Community facility delivery**				
Low			1	1
High			1.24(1.07,1.45)	1.09(0.94,1.26)
**Random parameters and model comparison**				
Community-level variance	0.48(0.37,0.61)	0.38(0.29,0.50)	0.44(0.34,0.56)	0.36(0.27,0.47)
ICC (%)	12.73	10.59	11.66	9.86
MOR (%)	1.93	1.81	1.87	1.76
PCV (%)	Reference	18.75	8.34	25.00
LLR	-8283.54	-8114.76	-8245.91	-8090.84
DIC	16,567.08	16,229.52	16,491.82	16,181.68
AIC	16571.08	16271.76	16503.81	16245.69

Note: * significant at *p*-value < 0.05, ICC = Intra cluster correlation, MOR = Median Odds Ratio, DIC = Deviance information criterion, LLR = Log Likelihood Ratio, AIC = Akaike information criterion

## Discussion

The first two years of a child’s life are critical, and appropriate nutrition during this time is critical for the child’s healthy growth and development. Studies showed that incorrect food consumption and practices have a negative impact on the nutritional health of babies and children [26, 27]. The current study looked at how MMNPs affected the nutritional status of East African newborns during a 6- to 23-month period. Only 6.45% (95% CI, 6.19,6.72) of children aged 6–23 months in Eastern Africa had received any MMNPs from the recommended sources, according to the study. Previous studies conducted in African countries have reported higher rates of MMNPs consumption compared to the findings of the current study. For example, in Rwanda (2021), 38% of mothers added MMNPs to their children’s foods [28]. Similarly, in Madagascar (2017), the proportion was 48.3% [29], while in Nigeria (2022), it was approximately 76.7% [30]. These variations highlight the differences in MMNPs consumption rates across countries and contexts in Africa. Factors contributing to these differences include variations in healthcare systems, nutritional awareness, availability and accessibility of MMNPs, cultural practices, and socioeconomic factors [31]. The contrasting results between the current study and previous research emphasize the importance of conducting country-specific investigations to accurately understand the factors influencing MMNPs consumption. This underscores the need for tailored interventions and strategies that are contextually appropriate for each country to improve MMNPs utilization. Further research is needed to explore the reasons behind the disparities in MMNPs consumption rates and identify successful interventions and best practices from countries with higher consumption rates. Sharing experiences and lessons learned between countries can facilitate knowledge exchange, inform policies, and guide programs aimed at increasing MMNPs intake among children in East Africa.

Women’s occupational status, the mothers’ age, ANC, place of delivery, residence, wealth index, media exposure, birth interval, number of health visits in the previous 12 months, twin status, and residence were significantly associated with MMNPs intake status among children aged 6–23 months after controlling for individual and community-level factors. In this study, higher odds of MMNPs intake were observed among children whose mothers are older compared to those whose mothers are young women. There could be various reasons why older women did not give their children multiple vitamin supplements. One probable explanation is a lack of understanding regarding the benefits and availability of these powders [1, 27]. Many mothers are unaware that several micronutrient powders consuming foods rich in vitamins can help reduce or prevent micronutrient deficiencies, and improve their children’s general health and well-being. Another factor could be the lack of availability or price of different MNs [1, 27, 32]. The cost and availability of these powders in resource-constrained situations may make it difficult for young women to provide them to their children. Furthermore, access to healthcare facilities or distribution channels where these powders are available may be difficult. Cultural beliefs and traditions may also influence children’s acceptance or rejection of several micronutrient powders [33]. It is crucial to note that these causes may differ depending on the context and demographic. To ensure effective implementation and adoption, efforts to promote and increase the usage of various micronutrient powders should take these elements into mind.

Mothers who had given birth to multiple children and had a birth interval of more than 59 months had a higher tendency to practice MMNPs on their children than mothers who had given birth to single children and had a birth interval of less than 24 months. Although the we did not find similar literature, several reasons could be given for this related to maternal replenishment: The longer delivery gap gives women more time to recuperate and replenish their nutrient stores, lowering the likelihood of maternal depletion [34]. Mothers may have better nutritional health and be more likely to give their children MMNPs to ensure enough nutrition if they have more time between pregnancies. Mothers who have had numerous children may have accumulated more expertise and information about child nutrition over time [34]. They may be better aware of the benefits of MMNPs and the importance of feeding their children critical nutrients. All these might enhance their access to health care due to their multiple births and use of contraception.

When compared to their counterparts, children living in rural regions had lower odds of consuming MMNPs. A thorough investigation in Ethiopia and DHS findings show that urban residents had a higher likelihood of MMNPs ingestion than rural residents [35, 36]. The likely explanation could be related to gaps in information, skills, and attitudes, as well as food fortification and supplementation targeting urban rather than rural areas through community-based maternal and child health outreach initiatives. Mass media exposure can dramatically raise awareness and knowledge about the benefits of using MMNPs for children’s nutrition [37, 38]. Mass media can convey crucial information on the importance of MMNPs in avoiding nutritional deficiencies through ads, educational initiatives, and public service announcements [37–39]. Media has the capacity to shape individuals’ views on MMNPs by influencing their perception of social norms [38, 39]. When mass media campaigns include testimonies and experiences of successful MMNPs use, a positive social norm around supplying MMNPs to children can be established. Mass media can influence behavior change by promoting the use of MMNPs.

In this study, children with institutional delivery had higher odds of MMNPs intake than those whose mothers did not give birth anywhere other than a health facility. This outcome was consistent with earlier research conducted in Ethiopia [36, 40]. One possible explanation is that women who had ANC follow-up and gave birth at health facilities may have had the opportunity to get information, education, knowledge, and counseling from health experts. Caregivers may have learned about MMNPs supplementation through their ANC follow-up. Another argument could be that mothers with follow-up and facility deliveries reside closer to health care facilities and have more time or money to attend ANC. Furthermore, a systematic review and meta-analysis of dietary diversity feeding practices conducted in 2018 reveals that children whose mothers had ANC follow-up have a higher probability of eating diverse food than their contemporaries [35]. Similarly, children whose mothers had frequent interactions with health facilities were much more likely than others to have high MMNPs provision for their children. Furthermore, visits to health-care institutions may rise, which benefits moms’ skills and knowledge [41]. Other studies have found that following the advice of a health professional, in this case a mother, has a positive effect on iron supplementation for their children [42, 43].

According to this study, MMNPs powders intake was lower in children whose mothers worked and in the wealthiest homes than in children whose mothers did not work and in the poorest households. We discovered that the poorest people were substantially more likely to have strong adherence. It is possible that this is due to economically deprived populations being more sensitive to health messages and instructions. Alternatively, the rich are unconcerned about orders from health-care providers who may not be from the same social or economic class. Similar behavior has been observed with exclusive breast feeding, which is similarly more common among the poor than among the wealthy [41, 44]. They also have all they need to provide for their children rather than purchasing powders.

### Strength and limitations of the study

Finally, the key strengths of the study are its representativeness, large sample size, and availability of individual and community-level factors. This study used a multilevel modeling technique to provide a more reliable result that takes the hierarchical nature of the survey data into consideration. The DHS methodology also allows for comparison with various situations. However, the study also has limitations. For example, the mothers may have experienced recollection bias towards their child in the six months preceding the study. Conducting geographical distribution studies across countries with other factors and study types will provide a better understanding of the details.

## Conclusions

In this study, the overall consumption of MMNPs was lower than the national and international recommendations. After controlling for individual and community-level factors, women’s occupational status and age, ANC, place of delivery, residence, wealth index, mass media exposure, preceding birth interval, number of health visits in the previous 12 months, twin status, and residence were significantly associated with MMNPs intake status among children aged 6–23 months in the final fourth model in East Africa. Improving the quality and coverage of maternity, reproductive, and child health services such as ANC, PNC, family planning, institutional delivery, and utilizing the possibility of mother visitation to the health facility will have a greater potential for raising MMNPs among young children in East Africa. Using mass media to promote maternal and child feeding practices, as well as assisting economically disadvantaged and rural mothers, will all contribute to MMNPs.

### They way forward recommendations

#### To countries

##### Increase awareness and education

Governments should prioritize public health campaigns to raise awareness about the importance of consuming MMNPs among children aged 6–23 months. This can be achieved through targeted messaging in healthcare facilities, community outreach programs, and mass media channels.

##### Strengthen ANC services

Efforts should be made to enhance ANC services, ensuring that pregnant women receive appropriate counseling on the benefits of MMNPs for their children’s nutrition. ANC visits should also focus on educating women about the importance of continued MMNPS consumption during the complementary feeding period.

##### Improve access and availability

Governments should work towards ensuring the widespread availability and accessibility of MMNPs in healthcare facilities and community settings. This can be achieved through effective supply chains, distribution networks, and collaboration with relevant stakeholders.

#### To national and international agencies

##### Support research and monitoring

International agencies should encourage and support further research on MMNPs consumption, specifically in the East African region. This includes monitoring the prevalence and trends of MMNPs intake among under-five children to identify gaps and design targeted interventions.

##### Strengthen collaboration

International agencies can collaborate with national governments and local organizations to develop and implement comprehensive nutrition programs. This includes integrating MMNPs distribution and promotion into existing maternal and child health programs.

#### To health systems, and healthcare providers

##### Enhance counseling and support

Healthcare providers, including physicians, nurses, and community health workers, should receive training and guidance on counseling mothers about MMNPs consumption during ANC and postnatal care visits. They can play a vital role in providing accurate information, addressing misconceptions, and emphasizing the importance of MMNPs in a child’s nutritional needs.

##### Monitor and track MMNPs usage

Healthcare providers should incorporate monitoring and tracking systems to assess MMNPs usage among children during routine healthcare visits. This data can help identify areas of improvement and guide targeted interventions.

## Data Availability

All data concerning this study are accommodated and presented in this document. The detailed data set can be freely accessible from the www.dhsprogram.comwebsite.
